# Fatal case of newborn Lassa fever virus infection mimicking late onset neonatal sepsis: a case report from northern Nigeria

**DOI:** 10.1186/s40249-020-00731-1

**Published:** 2020-08-10

**Authors:** Taofik Oluwaseun Ogunkunle, Surajudeen Oyeleke Bello, Chinwe Immaculata Anderson, Rashida Musa, Rasaq Olaosebikan, Abdulazeez Imam

**Affiliations:** 1 Department of Paediatrics, Dalhatu Araf Specialist Hospital, Lafia, Nassarawa Nigeria; 2 Department of Pharmacology and Experimental Therapeutics, Thomas Jefferson University, Philadelphia, PA USA; 3 Department of Vaccines and Immunity, Medical Research Council Unit The Gambia at London School of Hygiene and Tropical Medicine, Atlantic Boulevard, Fajara, P.O. Box 452, Banjul, Gambia

**Keywords:** Lassa fever, Neonatal sepsis, Newborn, Newborn mortality, Nigeria

## Abstract

**Background:**

Lassa fever is a zoonotic viral infection endemic to the West Africa countries. It is highly fatal during pregnancy and as such reports of neonatal onset Lassa fever infections are rare in scientific literature. We report a fatal case of Lassa fever in a 26-day-old neonate mimicking the diagnosis of late-onset neonatal sepsis.

**Case presentation:**

The patient is a 26-day-old neonate who was admitted with a day history of fever, poor feeding, pre-auricular lymphadenopathy and sudden parental death. He was initially evaluated for late onset neonatal sepsis. He later developed abnormal bleeding and multiple convulsions while on admission, prompting the need to evaluate for Lassa fever using reverse transcription polymerase chain reaction (RT-PCR). He died 31 h into admission and RT-PCR result was positive for Lassa fever.

**Conclusions:**

Neonatal Lassa fever infection is highly fatal and can mimic neonatal sepsis. High index of suspicion is needed particularly for atypical presentations of neonatal sepsis in Lassa fever endemic areas.

## Background

Lassa fever is an acute viral haemorrhagic illness endemic to the West African sub-region and first discovered in Northern Nigeria [1]. The disease is transmitted to humans via contact with food or other items contaminated with urine or faeces from the natural reservoir, rodents [2]. *Mastomys natalensis* (natal multimammate mouse) was initially thought to be the only known reservoir, there is however evidence in literature to suggest other rodent species, for example, *Mastomys erythroleucus (*Guinea multimammate mouse*), Hylomyscus pamfi (*African wood mouse*) and Mus baoulei (the pygmy mice)* [3*,* 4]*.* Human-to-human transmission also occurs through contact with infected persons secretions or through vertical transmission from mother to child [5]**.**

The disease has an incubation period of 6 to 21 days and is symptomatic in about 20% of infected persons, who commonly present with symptoms similar to other more common tropical ailments such as malaria and typhoid fever [6]. Lassa fever is also highly fatal to the developing foetus and fatality rates as high as 87% have been described in fetuses and neonates with vertically acquired Lassa fever infection [7]. As such, description of neonatal onset Lassa fever infection is scarce in the literature.

Nigeria has experienced repeated Lassa fever outbreaks and is currently experiencing an epidemic with 979 recorded cases and a case fatality rate of 19.2% as of 26 April, 2020 [8]. In this report, we present a unique case of fatal Lassa fever infection in a 26-day-old baby admitted to the special care baby unit of a referral tertiary health center in North-Central Nigeria with an initial diagnosis of late-onset neonatal sepsis. Our report emphasizes the importance of a high index of suspicion for Lassa fever in atypical presentations of neonatal sepsis especially during outbreaks.

## Case presentation

The case is a 26-day-old term male neonate who was brought to the neonatal unit of the Dalhatu Araf Specialist Hospital by his maternal grandmother with complaints of high grade continuous fever, poor feeding and facial swelling which were noticed a day prior to presentation. The baby was said to have been delivered at home. Both the baby’s parents had died about a week earlier from a febrile illness spanning about 2 weeks and the grandmother initially admitted to having limited knowledge regarding the exact nature of their illness. The family had resided in a rural part of town and was of low socio-economic status.

On initial physical examination, the infant was fully conscious but febrile with an axillary temperature of 38 °C and weighed 2.8 kg. He was pale and had a left-sided pre-auricular lymphadenopathy with pustular rashes around his perineal area. He was not jaundiced and was not in respiratory distress. An initial diagnosis of late onset neonatal sepsis was made, and the patient was prescribed empirical antibiotics – intravenous cefotaxime (50 mg/kg, 12-hourly) and ampicillin/cloxacillin combination (50 mg/kg, 6-hourly). This was based on the local unit protocol for babies admitted from the community. His bedside random blood sugar check was normal (7.4 mmol/L) and a packed cell volume was 30%. His samples were sent for full blood count and blood culture **(**Table 1**).**

**Table 1:** Laboratory investigation results of index case

Laboratory investigations	Values/Results	Reference ranges/remark
^a^**Complete blood count**		
White cell count	2300 per mm^3^	4000–10 000 per mm^3^
Neutrophils	38% (874 per mm^3^)	
Lymphocytes	57% (1311 per mm^3^)	
Platelet	179 000 per mm^3^	150 000–400 000 per mm^3^
Packed cell volume	30%	> 35%
Blood film appearance	Hypochromic, microcytic	
**Blood culture**	No growth after 7 days of incubation	
**HIV screening**	Negative	
**Random blood sugar readings**	5.6 mmol/L, 7.4 mmol/L, 10.6 mmol/L	Normal

^a^Sample obtained at presentation

**Fig. 1:**
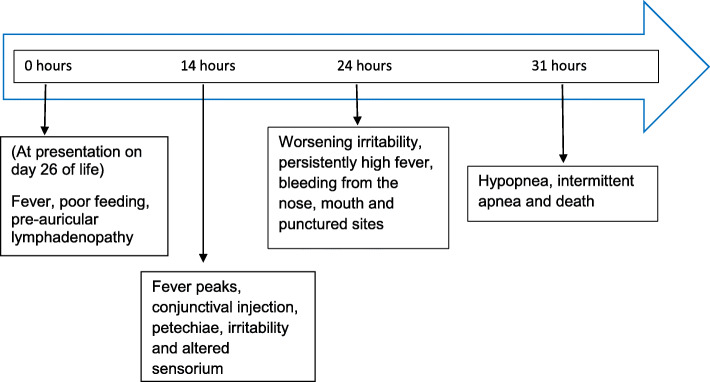
Timeline for index case clinical presentation

About 14 h into admission, his fever peaked at 39.4 °C, and he subsequently experienced multiple convulsive episodes with profound irritability. He was later noticed to have developed a hyperemic conjunctiva with facial, neck and truncal petechiae. A timeline of his symptoms is detailed in Fig. 1. As a result of the progress of his symptoms, the admitting team considered the possibility of meningitis and disseminated intravascular coagulopathy and suspected Lassa fever due to an existing outbreak in the state. Intravenous cefotaxime was then substituted with ceftriaxone (100 mg/kg/day) due to better cerebrospinal fluid penetration of the latter. He was also started on intravenous phenobarbitone for seizure control. On further direct questioning of his maternal grandmother, she revealed that the baby’s mother had died from a bleeding associated febrile illness around the time of delivery while his father also died few days before the mother’s demise from a similar illness. A blood sample was sent to the Nigerian Centre for Disease Control national reference laboratory in Abuja, located in a neighbouring state. The baby was isolated and transfused with fresh whole blood and stringent infection prevention protocol was activated. He subsequently developed shallow breathing and intermittent apnea and died about 31 h into admission despite active resuscitation. Virology report using reverse transcription polymerase chain reaction (RT-PCR) was made available 48 h after the baby’s demise and was positive for Lassa fever virus.

The epidemiology unit of the hospital commenced contact tracing and identified close contacts of the index case. All contacts (health workers and family members) were stratified into three risk category according to the National guideline on Lassa fever case management [9]. Seven persons were categorized as high risk; two doctors, three nurses and two health attendants and they were all commenced on oral ribavirin for post-exposure prophylaxis using the Irrua regimen (100 m/kg in 2 divided doses on day 1, then 25 mg/kg/day on day 2 to 7, and 12.5 mg/kg on day 8 to 10) [9]. Three out of the seven high risk contacts completed the seven-day therapy while the rest stopped the medication after day two to three of therapy due to experience of side effects. None of the ‘no risk’ and ‘low risk’ category developed symptoms during the 21 days of observation and none of the ‘high risk’ contact was positive for Lassa fever virus.

## Discussion and conclusions

The Lassa fever virus is an RNA virus from the Arenaviridae family. Lassa fever, although has been in existence for decades but still has no vaccine. Geographically the disease is endemic to West African countries and outbreaks have occurred in Nigeria, Guinea, Liberia and Sierra Leone, where the disease is endemic [6]. Imported cases to the United States and Europe have also been described in literature [10]. Risk factors for acquiring the virus include residence in rural areas or communities with poor sanitation, crowded living conditions, family contacts of infected persons and health workers [2, 11].

Lassa fever affects all age groups but the clinical course of paediatric Lassa fever is poorly described in literature [12]. Available evidence suggests paediatric illness presents with an acute febrile illness, generalized edema, abdominal distension, and bleeding [13]. In other literature, paediatric patients with Lassa fever have also been shown to present with acute abdomen, convulsive episodes or in severe disease, clinical features of a swollen baby syndrome which is characterized by widespread edema, abdominal distention, and bleeding [14–16]. There is a dearth of knowledge of clinical course for neonates with Lassa fever virus infection because of an increased risk of adverse pregnancy outcomes in vertically acquired infection [17]. Moreover, typical adult and older paediatric age symptoms such as malaise, sore throat, retrosternal pain and myalgia are impossible to elicit in neonates.

In the index case, high grade fever, lymphadenopathy, convulsions and uncontrolled bleeding were consistent with the existing symptoms described in older paediatric populations [13, 16]. In addition, the baby presented with an atypical onset of symptoms at the 25th day of life which is outside the known maximum incubation period of 21 days. The managing team initially made a diagnosis of late-onset neonatal sepsis (LONS) and following onset of bleeding and convulsions, considered well known complications of LONS such as disseminated intravascular coagulation (DIC) and meningitis. The diagnosis of Lassa fever was explored due to the current national Lassa fever epidemic and suspicious death of both the neonates parents shortly after delivery. This has important implications, as Lassa fever is hyper-endemic in Nigeria and can present in the absence of epidemics. In addition, to observing universal precaution for all patients, clinicians and health workers attending to cases of neonatal sepsis might need a higher index of clinical suspicion particularly in neonates presenting with complications of DIC and meningitis or a history of sudden parental death following delivery. This underscores the importance of taking a detailed medical history and being familiar with local clinical case definitions in resource-poor settings and also taking necessary steps when these clinical case definitions are met. Early exploration of the cause of death of parents of the index case might have helped in early diagnosis and treatment, as the parents’ clinical history of death following a febrile illness with bleeding already provided adequate case definition for Lassa fever irrespective of an epidemic. Nevertheless, the high index of suspicion prevented potential for nosocomial acquired Lassa fever among health workers and other patients. Nosocomial outbreaks of Lassa fever have been frequently reported in West Africa due to poor adherence to infection control practices and standard universal precaution measures [13].

Results of RT-PCR for the index case was obtained 48 h after demise as the RT-PCR sample had to be sent to a national reference laboratory several kilometers away. This highlights challenges faced by health professionals in real-time treatment and management of Lassa fever in endemic resource-poor settings and suggests the need for further capacity strengthening and decentralization of Lassa fever testing. In the short to medium term however, research and development might need to focus on development of rapid testing kits for Lassa fever that can be used as point-of-care before confirmation at regional molecular laboratory level.

Our reported case resulted in fatality, but there is some evidence from a case report and a retrospective cohort study to suggest positive newborn outcomes in few pregnant mothers [17, 18]. In these cases, Lassa fever was diagnosed early and treated in the third trimester of pregnancy and these were reports of mothers who delivered in specialist or tertiary-level health facilities [17, 18]. The mother of our index participant delivered in at home and was not identified as a probable Lassa fever case. This raises a key issue about knowledge on symptomatology of Lassa fever among the general populace in Nigeria and this might need to be addressed by local health policy makers.

Neonatal Lassa fever is highly fatal and when presenting in the neonatal period might be indistinguishable from neonatal sepsis. A high index of clinical suspicion and detailed histories are crucial in neonatal Lassa fever diagnosis in endemic settings and this should be entertained even in the absence of outbreaks. Improving capacity for laboratory identification of the virus is important for early diagnosis and treatment in these settings. Public enlightenment programs on Lassa fever are need to educate the general public on clinical recognition of Lassa fever.

## Data Availability

All data relating to this study are presented within the manuscript. Other materials are available from the corresponding author upon reasonable request.

## Abbreviations

DIC: Disseminated intravascular coagulation
LONS: Late onset neonatal sepsis
RT-PCR: Reverse transcription polymerase chain reaction
